# Meeting of the New York State Medical Society

**Published:** 1871-01

**Authors:** 


### Meeting of the New York State Medical Society.

The Medical Society of the State of New York will hold its next Annual Session on the first Tuesday of February, (Feb 7th,) in the city of Albany. It is to be hoped that there will be a general attendance, and that those who do attend may present, or be able to listen to, well considered, well written papers upon the medical subjects of the times.

The transactions of this society have thus far been very creditable to it; and we have no doubt many valuable contributions will be, this year, offered, as the interest in this society appears to be constantly increasing. Some of the very best papers of the season appear first before our State Medical Society. There are various legislative topics also to be presented; and we bespeak for the Society a meeting of unusual interest.

